# Supplementary material for: Functional Outcomes of Cochlear Implantation in Children with Bilateral Cochlear Nerve Aplasia
Source: Medicina (Kaunas). 2022 Oct 17;58(10):1474. doi: 10.3390/medicina58101474 (PMC9607978; doi:10.3390/medicina58101474)
Supplement: Supplementary file 1 [file medicina-58-01474-s001.zip › medicina-1959362-supplementary.pdf]

Supplementary Table S1. Details of operative data in pediatric subjects with bilateral cochlear aplasia.

Subject No.	Age (Months)	Sex	laterality	approach	electrode	Cochlear anomaly
1	12	F	R	RW	Clarion (AB)	B) cochlear hypoplasia
2	19	M	B	RW	Flex 24 (Medel)	
3	11	F	B	RW	CI 522 (Cochlear)	
4	27	M	L	RW	Contour (Cochlear)	B) cochlear hypoplasia
5	16	M	L	RW	Contour (Cochlear)	R) lateral semicircular canal dysplasia
6	17	M	B	RW	Flex 24 (Medel)	
7	12	M	B	RW	CI 522 (Cochlear)	B) cochlear hypoplasia
8	14	F	B	RW	Flex 24 (Medel)	
9	24	M	B	RW	CI 512 (Cochlear)	
10	22	F	B	Cochleostomy	Synchrony (Medel)	
11	37	M	R	RW	CI 422 (Cochlear)	
12	15	F	L	RW	Contour (Cochlear)	B) lateral semicircular canal dysplasia
13	25	M	B	RW	Contour (Cochlear)	B) vestibule and semicircular canal dysplasia
14	13	F	B	Cochleostomy	CI 532 (Cochlear)	
15	10	M	B	Cochleostomy	CI 522 (Cochlear)	B) cochlear hypoplasia<br>B) bulbous dilatation of lateral semicircular canal
16	28	F	B	RW	Flex 28 (Medel)	
17	15	F	B	RW	CI 422 (Cochlear)	
18	13	F	B	Cochleostomy	CI 532 (Cochlear)	L) semicircular canal dysplasia
19	17	M	B	RW	CI 632 (Cochlear)	
20	12	F	B	RW	CI 522 (Cochlear)	
21	13	F	B	Cochleostomy	CI 532 (Cochlear)	

Abbreviations: F, female; M, male; R, right; L, left; B, bilateral; RW, round window.
